# Begomovirus capsid proteins interact with cyclic adenosine monophosphate (cAMP)-specific phosphodiesterase of its whitefly vector and modulate virus retention within its vector

**DOI:** 10.1128/jvi.02172-24

**Published:** 2025-02-11

**Authors:** Saptarshi Ghosh, Banani Mondal, Ola Jassar, Murad Ghanim, Saurabh Gautam, Vamsidhar Reddy Netla, Rajagopalbabu Srinivasan

**Affiliations:** 1 Department of Entomology, University of Georgia 166833 , Griffin, Georgia, USA; 2 Department of Entomology, The Volcani Center, Agriculture Research Organization, Rishon Lezion, Israel; 3 Robert H. Smith Faculty of Agriculture, Food and Environment, Hebrew University of Jerusalem 72256 , Rehovot, Israel; 4 Alliance of pest control districts, Tulare, California, USA; Iowa State University, Ames, Iowa, USA

**Keywords:** whitefly protein, begomovirus, interaction, validation, transmission

## Abstract

Begomoviruses are whitefly-transmitted ss-DNA viruses that infect dicotyledonous plants and contribute to major economic losses to global crop production. Invasion and establishment of an aggressive cryptic species of *Bemisia tabaci*, known as the B cryptic species, has severely constrained vegetable production in the southeastern and southwestern United States. Disruption of genes/pathways critical for whitefly-mediated transmission can be effective for the management of begomoviruses. In this study, yeast two-hybrid (Y2H)-based screening of *B. tabaci* cDNA library identified a cyclic adenosine monophosphate (cAMP)-specific phosphodiesterase-4 (PDE4) of the whitefly as an interacting partner with capsid proteins (CPs) of old- and new-world begomoviruses. Interactions of PDE4 with begomovirus CPs were validated by glutathione-S-transferase (GST) pull-down assay and co-immunolocalization in whitefly midgut. The PDE4 family of enzymes hydrolyzes cAMP and regulates intracellular cAMP levels. This study conclusively proves that acquisition of begomoviruses downregulates the expression of PDE4 (mRNA and protein) resulting in elevated cAMP levels within the whitefly. The role of cAMP post virus acquisition is further elucidated wherein elevation of cAMP by chemical inhibition or gene (PDE4) silencing resulted in increased retention and transmission of begomoviruses. Similarly, decreased cAMP levels resulted in reduced begomovirus retention. The results of this study demonstrate that whitefly-mediated transmission of begomoviruses is regulated by intracellular cAMP by unknown mechanisms.

**IMPORTANCE:**

Begomoviruses, transmitted by the sweetpotato whitefly (*Bemisia tabaci* Gennadius), are the causal agents of many economically important plant virus diseases. Lack of host plant resistance against begomoviruses, high whitefly abundance, and whitefly’s ability to develop insecticide resistance rapidly often render the commonly used management practice ineffective. This study demonstrates how begomovirus retention within whitefly and its transmission can be modulated by altering cyclic adenosine monophosphate (cAMP) expression of its insect vector. Naturally occurring bio-pesticides that target insect cAMPs are known. Our findings can lead to alternative strategies for the management of begomoviruses by targeting whitefly cAMP using chemicals, botanicals, or RNAi-based insecticides.

## INTRODUCTION

The sweetpotato whitefly, *Bemisia tabaci* Gennadius, is a cryptic species complex (1). *B. tabaci* is infamous for its ability to transmit several economically important plant viruses that challenge food security in the tropics and semi-tropics (2, 3). Among the diverse group of viruses transmitted by *B. tabaci*, ss-DNA viruses with geminate particles belonging to the genus *Begomovirus* (family *Geminiviridae*) constitute the largest group (2). These viruses are transmitted by *B. tabaci* in a circulative and non-propagative mode. The begomovirus genomes exist in either monopartite form with a single circular ss-DNA (2.8 kb) known as DNA-A or bipartite form with an additional single circular ss-DNA (~2.8 kb) known as DNA-B (4). AV1 gene in DNA-A encodes the highly conserved capsid protein (CP), which is recognized specifically by cell surface receptors in whitefly midgut and salivary gland tissues, resulting in stringent virus-vector relationships (2). While circulating within the whitefly, begomoviruses interact with multiple intracellular proteins and bacterial endosymbionts through CP, which facilitates tropism across tissue barriers such as the midgut, salivary glands, and the hemolymph (2).

Begomoviruses are broadly classified as new world or old world based on their origin, and distinguished by the absence of AV2 gene in the DNA-A component of the former. Increased agricultural trade between the new and old world in the past decades has led to exchange and establishment of exotic begomoviruses and invasive whitefly cryptic species in non-native geographical areas (5). A highly invasive cryptic species of *B. tabaci,* B cryptic species (also known as Middle East Asia Minor MEAM1), invaded the USA in the late 1980s and established itself across the southeastern and southwestern states, and displaced the indigenous whitefly cryptic species “A” (6–8). In these regions within the United States, B cryptic species of *B. tabaci* constrains vegetable production by efficiently transmitting new-world begomoviruses such as cucurbit leaf crumple virus (CuLCrV) that infects cucurbit crops and sida golden mosaic virus (SiGMV) that infects snap bean (9, 10). The B cryptic species also efficiently transmits old-world viruses such as tomato yellow leaf curl virus (TYLCV) that infects tomato (5, 9, 11). Management of begomoviruses is challenging due to the limited availability of host plant resistance (12). Consequently, there is a heavy reliance on cultural and chemical practices (13, 14). However, extremely high whitefly abundance during some seasons (for instance, fall season in the southeastern US) renders insecticidal management of virus transmission ineffective (14–17). There is a dire need for novel and sustainable strategies for the management of whitefly-transmitted begomoviruses in vegetable production. Thus, identification of whitefly proteins/pathways, involved in the transmission of begomoviruses, is important for the conception of novel virus management strategies. Although multiple whitefly proteins with inhibitory roles (18–22) against begomoviruses have been identified, understanding of insect defense against viruses is still far from complete and yet to be exploited to reduce virus transmission.

This study used yeast two-hybrid (Y2H)-based screening of *B. tabaci* (B cryptic species) cDNA library with the CP of CuLCrV as bait and identified cyclic adenosine monophosphate (cAMP)-specific 3’,5’-cyclic nucleotide phosphodiesterase (PDE) of the whitefly as an interacting partner. The cAMP-specific phosphodiesterases hydrolyze cAMP, an intracellular second messenger of extracellular ligand action (23), and play an important role in modulating inflammatory responses (24). In this study, cAMP levels were elevated and diminished within the whitefly using chemical inhibitors and gene silencing. This study also demonstrated that the retention and transmission of begomoviruses are directly dependent on intracellular cAMP levels. These outcomes have important implications for the management of epidemics of begomoviruses.

## MATERIALS AND METHODS

### Maintenance of *B. tabaci* (B cryptic species) population and virus isolates

*B. tabaci* (B cryptic species) was maintained on cotton plants inside insect-proof cages at 25 ± 6°C, 60% relative humidity (RH), and 14L:10D (17). CuLCrV, TYLCV, and SiGMV virus isolates were maintained using whitefly-mediated inoculation in squash (cv. F1 Goldstar hybrid), tomato (cv. Florida 47), and sida, respectively (10, 15, 17).

### Construction of *B. tabaci* (B cryptic species) cDNA library and Y2H screening

Total RNA (2 µg) extracted from *B. tabaci* adults was used for the synthesis of first-strand cDNA utilizing the Make Your Own Mate & Plate Library System with CDS III Adaptor Primer and Smart III Oligo (template switching) as per manufacturer’s instructions (Takara Bio, San Jose, CA, USA). The first-strand cDNA was used as template for long distance PCR with 5´ and 3´ primers, purified using chroma spin + TE 400 columns, and co-transformed into competent Y187 yeast cells for gal4 AD-*B. tabaci* B cDNA library construction as previously described (25). The pGBKT7 construct with CuLCrV CP insert (5) was transformed in Y2H gold strain of yeast and screened against the *B. tabaci* B library on (–ALTH) minimal medium (5). Prey plasmids from colonies developing blue color on X-alpha-gal supplemented –ALTH medium were recovered and sequenced from both directions by Sanger sequencing. Nucleotide sequences were translated *in silico* in frame with the GAL4 activation domain and annotated using BLASTp, CDD, and Pfam databases. PDE4 gene starting from ATG was PCR amplified using specific primers (Table 1) from cDNA of B cryptic species and cloned into pGADT7 and was screened against the CPs of new-world viruses (CuLCrV, SiGMV), old-world virus (TYLCV), and empty pGBKT7 by one-to-one mating assays (5) on –ALTH medium and lesser stringent –LTH minimal medium, respectively.

**TABLE 1:** Primers used in this study^*a*^

Sequence (5´→3´)	Target gene	Length	Objective
F-GGAGGCCAGTGAATTC **ATG**CAAGCGGAACAAGGTAGTATC R-CGAGCTCGATGGATCC **TCA**CATCTGTGCAGTTGAGTCCTC	PDE4 (*B. tabaci*)	2,016 bp	PCR amplification of PDE4 for Gibson cloning into pGBKT7
F- TGACATGGTGCTTTCAACGG R- GCGGTTTCGTTGGGTTACTT	PDE4 (*B. tabaci*) (Bta15489)	182 bp	qPCR of PDE4
F-*GTGGAATTC* AGTTCAAAAGAATGCTCAATAAG R-*GTGGGATCC* GAGAACATTAGTAGACTGAGT	PDE4 (*B. tabaci*) (Bta15489)	557 bp	Cloning into TRV2 for PDE4 silencing
F-*GTGGAATTC* CAACAGGAACAACTCCTCCT R-*GTGGGATCC* GGACTCCATGTGGTTTGCTA	Adenylyl cyclase (*B. tabaci*) Bta07006	540 bp	Cloning into TRV2 for AC silencing
F- TTGATGCTCCGGTTCTTGAG R- ACCAATCAAGGTGACGACAG	Adenylyl cyclase (*B. tabaci*) Bta07006	195 bp	qPCR of AC
F-GGTT*GCGATCGC*C **ATG**TCGAAGCGACCAGGC R-GTGT*GTTTAAAC*CTA **TTA**ATTTGATATTGAATCATA	TYLCP-CP		Cloning into pFN2A for N-GST-fused CP
F-GGTT*GCGATCGC*C **ATG**CCGAAGCGCGATGCCCCAT R-GTGT*GTTTAAAC*CTA **TTA**ATTTGTTATCGAATCATA	CuLCrV-CP		-do-
F-GGTT*GCGATCGC*C **ATG**CCTAAGCGCGAATTGCCAT R-GTGT*GTTTAAAC*CTA **TTA**ATTCATAAGCGAATCATAG	SiGMV-CP		-do-
F-ATTCTCCACGTGGAGGTCCT R-TGGTGGTTGTAGACTACGTG	AV1 gene of CuLCrV	540 bp	PCR detection of CuLCrV

^*a*^ Bold ietters indicate start and stop codons. Italicized letters indicate restriction endonuclease enzyme sequences.

### Pull-down assay of PDE4 using glutathione-S-transferase (GST)-tagged CPs of CuLCrV, SiGMV, and TYLCV

CP genes of CuLCrV, SiGMV, and TYLCV were cloned in pFN2A vector (Promega) using *Sgf*I and *Pme*I restriction enzymes to express them as fused proteins with N-terminal GST tag. PDE4 gene was inserted in pRSET A (ThermoFisher Scientific, USA) using the *Bam*HI and *Eco*RI restriction sites to express it as a fused protein with N-terminal 6x-histidine tag. All the constructs were transformed to BL21 DE3 pLysS strain, and protein expression was induced with 0.4 mM isopropyl β-D-1-thiogalactopyranoside (IPTG) at 28°C for 5 hours. Bacterial lysates expressing fused GST-CuLCrV/SiGMV/TYLCV CP proteins and GST control were incubated with MagneGST particles (cat#V8600, MagneGST protein purification system, Promega, Madison, WI, USA) to immobilize the GST-tagged proteins as per manufacturer’s instructions. MagneGST particles immobilized with the respective GST-tagged proteins were incubated with bacterial lysates expressing fused 6x-his-PDE4 overnight at 4°C, and washed three times with 400 µL of binding/wash buffer followed by boiling with 35 µL of 1× Laemmli buffer. The proteins were electrophoresed on a 12% polyacrylamide gel and transferred to a nitrocellulose membrane; the membrane was cut into two pieces between 55 and 70 kD ladder bands followed by overnight incubation at 4°C with either monoclonal anti-histidine (1:2,000) (cat#MAB3834, Millipore Sigma, USA) or anti-GST (1:1,000) (cat#8-326, ThermoFisher Scientific) antibodies. Histidine-tagged PDE4 and GST-tagged CP proteins were detected chromogenically using anti-mouse secondary antibodies labeled with alkaline phosphatase (1:30,000) (cat#A3562, Millipore Sigma, Burlington, MA, USA) and Western Blue stabilized substrate for alkaline phosphatase (Promega, Madison, WI, USA).

### Immunolocalization of PDE4 and TYLCV in *B. tabaci* midgut

Viruliferous whitefly adults reared on TYLCV-infected tomato plants and non-viruliferous adults reared on cotton were dissected in phosphate-buffered saline (PBS), and the midguts were fixed in 4% paraformaldehyde for 30 minutes at room temperature followed by permeabilization with 0.1% Triton X-100 for 30 minutes at room temperature. The midgut samples were washed with PBST thrice, incubated for 1 hour in blocking buffer [PBST + 0.5% bovine serum albumin (BSA)] followed by overnight incubation with anti-TYLCV-CP polyclonal antibody (1:500) at 4°C. The midgut samples were washed thrice with PBST and incubated for 2 hours at room temperature with cy5-conjugated donkey anti-rabbit secondary antibody (Jackson ImmunoResearch Laboratories, PA, USA) diluted (1:1,000) in blocking buffer. The midgut samples were then washed thrice with PBST and incubated overnight at 4°C in blocking buffer containing anti-PDE4 polyclonal antibody (1:1,000). Subsequently, the midgut samples were washed thrice with PBST and incubated for 2 hours at room temperature with cy3-conjugated donkey anti-rabbit secondary antibody (Jackson Laboratories) diluted (1:1,000) in blocking buffer. The midgut samples incubated with cy3/cy5-conjugated secondary antibody without exposure to the anti-CP or anti-PDE4 antibody were used as negative control. Finally, the midgut samples were washed thrice with PBST and mounted with a buffer [PBS + 10 µg 4′,6-diamidino-2-phenylindole (DAPI)] on a glass slide with a cover slip, sealed using nail polish, and viewed under an Olympus IX81 laser scanning confocal microscope.

### Relative expression of cAMP phosphodiesterase (PDE4), quantitation of cellular cAMP post virus acquisition, and inhibition of PDE4

To know whether acquisition of begomoviruses can alter PDE4 expression, the levels of PDE4 (mRNA and protein) by qPCR and western blot and cAMP by enzyme-linked immunosorbent assay (ELISA) were compared between viruliferous (CuLCrV/TYLCV) and non-viruliferous whitefly adults. Whitefly adults were released on virus-infected (TYLCV/CuLCrV) or virus-free tomato/squash plants and collected at different time points (12, 24, 48 hours) post-acquisition. Relative expression of PDE4 mRNA was compared between viruliferous and non-viruliferous whitefly adult samples by qPCR. To further validate the findings, PDE4 protein expression was compared between viruliferous and non-viruliferous whitefly adults post 12 hours acquisition access period (AAP) on CuLCrV/TYLCV-infected and non-infected squash or tomato plants, respectively. Whitefly total protein was extracted from 100 female whitefly adults per replicate for the different treatments by CytoBuster Protein Extraction Reagent (cat# 71009-3, Millipore Sigma, USA). The extracted protein samples were quantified using Coomassie Protein Assay Reagent (cat#1856209, Thermo Scientific, USA). The PDE4 protein expression was measured by western blot as mentioned earlier; 30 µg total protein was loaded in each lane of 12% polyacrylamide gel, and the PDE4 level was detected using 1:5,000 dilution of a polyclonal anti-PDE4 antibody generated by immunizing two rabbits with a 243 amino acid long partial PDE4 protein (NDESVLENHHLVVEFKLLQKEGCDIFINLSKKQKQTLRKMVIDMVLSTDMSKHMSLLADLKAMVETKKVAGSGVLLLDNYTDRIQVLENLVHCADLSNPTKPLPLYRRWVDLLMEEFFQQGDKEREQNLDISPMCDRHSATIEKSQVGFIDYIVHPLWETWADLVHPDAQEILDMLEENRDWYQSMIPPSPPVNEGENRLDSDVEEGEESEPPNPNPPPVPQDSSIRFQVTLEEGDEDSTAQM) expressed in *Escherichia coli* (CUSABIO, Houston, Texas, USA). A goat anti-rabbit secondary antibody (1:30,000) conjugated to horseradish peroxidase (Cat#31480, Invitrogen, USA) and enhanced chemiluminescence substrate (cat# 1705062, BioRad, USA) were used for the visualization of protein bands. Monoclonal anti-human α-tubulin antibody (Cat#MA5-31466, ThermoFisher Scientific, USA) was used in 1:2,000 dilution in blocking solution for loading control. The volume (intensity) of the protein bands was quantitated using ImageJ (https://imagej.net/ij/) and the volumes of PDE4 bands were normalized against α-tubulin bands.

Cellular cAMP levels were compared between non-viruliferous and viruliferous (CuLCrV) whitefly adults. Whitefly adults post 12 hours of AAP on CuLCrV-infected and non-infected squash plants were collected and snap-frozen. Furthermore, cAMP was quantified from a pool of 50 female insects using the cAMP-specific ELISA kit as described below. Significance of differences of means of PDE4 expression and cAMP were analyzed using Student’s *t*-test.

Rolipram (cat#0905, Tocris Bioscience, Bristol, UK), a selective inhibitor of cAMP phosphodiesterase, was used to inhibit PDE4 and stimulate cellular cAMP within whitefly adults. The increase in cellular cAMP post feeding with rolipram was validated by quantitation of cAMP from adult whitefly samples using the Direct cAMP ELISA kit (cat#ADI-900-066A, ENZO Life Sciences, Farmingdale, NY, USA). Whitefly adults after 48 hours of feeding on 20% sucrose diets containing either 200 µM rolipram or 0.8% ethanol were collected and immediately snap-frozen in liquid nitrogen. Fifty females were homogenized in 250 µL of 0.1N HCl and kept on ice. An aliquot (25 µL) of this lysate was used for protein quantitation using Pierce Coomassie protein assay kit (cat#23200, ThermoFisher Scientific). The remaining lysate was centrifuged at 5,000 × *g* at 4°C to pellet debris. Two technical replicates, each with 100 µL of the supernatant, were used for estimation of cAMP in the samples alongside cAMP standards (non-acetylated) using the competitive ELISA kit as per manufacturer’s instructions. Sample cAMP concentrations (picomoles per milliliter) were obtained by fitting the OD values in the regression equation, which was generated by plotting the standards. The concentrations of cAMP were normalized to the protein content (picomoles per milligram proteins) in the lysate. Five separate biological replicates from each treatment were estimated for cAMP, and the means of four biological replicates (one outlier removed) were analyzed using Student’s *t*-test.

### Effect of rolipram on virus retention by whiteflies

Whitefly adults were fed on 20% sucrose diet containing either 200 µM rolipram (solubilized in ethanol) or 0.8% ethanol as control for 48 hours. The diet-fed whiteflies were allowed 24 hours of acquisition access to either CuLCrV-infected squash or TYLCV-infected tomato plantlets excised from a single infected source plant, followed by overnight gut clearing on cotton plants. Total DNA extracted from pools of 10 gut-cleared whitefly adults was used as template for relative quantitation of CuLCrV and TYLCV DNA retained using CuLCrV-qF/R and TYLCV-V2F/R and normalized to the β-tubulin gene of the whitefly using the ΔΔCt method (5). Significant differences of means were compared using one-way analysis of variance (ANOVA).

### Inhibition of adenylyl cyclase to decrease cellular cAMP and effect on virus retention

SQ22536 (cat#1435, Tocris Bioscience), an inhibitor of adenylyl cyclase (AC), was used to diminish cellular cAMP in the whitefly. Whitefly adults were fed for 48 hours on 20% sucrose diet containing 200 µM of SQ22536 (solubilized in ethanol) or 0.8% ethanol as control. The decrease in cellular cAMP post treatment with SQ22536 was validated by quantitation of cellular cAMP levels between SQ22536 fed and control whitefly adults by using the Direct cAMP ELISA kit (cat#ADI-900-066A, ENZO Life Sciences, Farmingdale, NY, USA). Sixty-five female whitefly adults were homogenized in 455 µL of ice-cold 0.1N HCl, and cAMP levels were quantitated using cAMP-specific ELISA kit according to the same protocol as mentioned in the earlier section. The significance of differences of cAMP levels were measured by Student’s *t*-test. To know the effect of reduced cAMP on begomovirus retention, whitefly adults fed on SQ22536-supplemented or control diet were allowed 24 hours of acquisition access on CuLCrV- or TYLCV-infected squash/tomato plantlets, gut cleared by overnight feeding on cotton plants, and subjected to quantitation of retained CuLCrV/TYLCV DNA by qPCR as described above. Significant differences of means were compared by one-way ANOVA.

### Virus-induced gene silencing of PDE4, AC, and effect on virus retention

To further validate the role of cAMP, virus-induced gene silencing using tobacco rattle virus-based vectors were used to silence PDE4 and AC genes in the whitefly as previously described (5, 26). Fragments of the PDE4 and AC genes (557 bp and 540 bp, respectively) (Table 1) were cloned into the multiple cloning site region of pTRV2 vector using *Eco*RI and *Bam*HI restriction enzymes. The PDE4_TRV2, AC_pTRV2, pTRV2 control, and pTRV1 were transformed separately into the LBA4404 strain of *Agrobacterium tumefaciens*, induced for 3 to 5 hours at room temperature. pTRV1 and PDE4_pTRV2 or AC_TRV2 or TRV2 control cultures were mixed in 1:1 proportion (vol/vol) and infiltrated within the leaves of 12-day-old tomato seedlings using a 1 mL syringe. Transformation of tomato plants was confirmed with detection of PDE4/AC mRNA on systemic leaves 12 days post inoculation by RT-PCR. *Bemisia tabaci* colonies were set up by releasing adult whiteflies (F0) on transformed plants for 10 days; after that, all F0 whiteflies were removed from plants. Relative expression of PDE4 and AC mRNA normalized to the whitefly β-tubulin mRNA in newly emerged F1 adult populations of whitefly feeding on plants inoculated with pTRV1 + PDE4/AC-pTRV2 constructs or TRV1 + TRV2 (control) constructs was quantitated by qPCR, and their means were compared by one-way ANOVA to confirm silencing. Following confirmation of silencing, F1 adults were given acquisition access to CuLCrV/TYLCV-infected squash/tomato plants for 24 hours, gut cleared by overnight feeding on cotton plants, and relative amounts of virus DNA retained was quantitated by qPCR. Significant differences of means were compared by one-way ANOVA.

### Effect of rolipram/SQ22536 on transmission of CuLCrV by *B*. *tabaci*

*Bemisia tabaci* adults (2–7 days old) reared on cotton plants were collected and provided with a 48 hour feeding access on 20% sucrose diet containing 200 µM of rolipram or 0.8% ethanol as control. Whitefly adults were then released separately onto a squash leaf excised from a single CuLCrV-infected squash plant for an AAP of 48 hours. Adults collected after acquisition access from the infected leaf were allowed life-long inoculation access (10 adults/plant) onto young squash seedlings (cv. F1 Goldstar hybrid 1-2 true leaf stage) in three separate replicated experiments. Total DNA was extracted from inoculated squash plants 15 days post inoculation and PCR indexed for CuLCrV infection using primers targeting the CP (AV1) gene (F-ATTCTCCACGTGGAGGTCCT, R-TGGTGGTTGTAGACTACGTG, 540 bp). Similarly, whitefly adults fed for 48 hours on 20% sucrose diet with and without 200 µM SQ22536 were provided with a 48 hour AAP and an inoculation access on non-infected cucurbit plants in two replicates containing 12 plants each. The plants were tested for CuLCrV 15 days post inoculation by PCR. Significant differences in transmission efficiency were inferred by analysis using χ^2^ test.

Plant samples confirmed infected with CuLCrV in the transmission assays were further used for absolute quantitation of CuLCrV using CuLCrV-QF/R primers and CuLCrV-hydrolysis probe (9). The means of virus copy numbers/ng of DNA template between rolipram/SQ22536 and control plants were analyzed using Wilcoxon rank-sum test.

## RESULTS

### Identification of PDE4 as an interacting partner with CPs of begomoviruses

**Fig 1:**
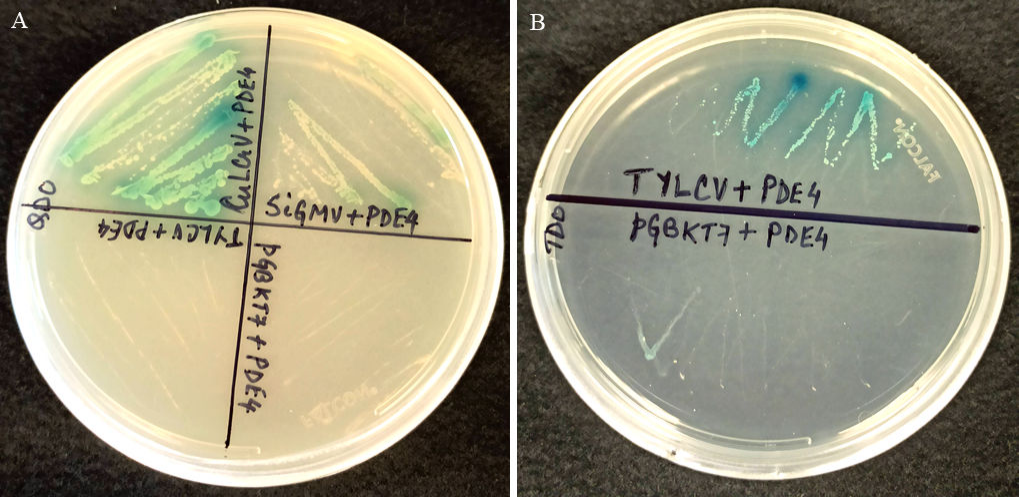
Interaction of PDE4 with CP proteins of CuLCrV, SiGMV, TYLCV, and empty pGBKT7 by one-to-one Y2H mating assays on (**A**) stringent −ALTH and (**B**) less stringent −LTH minimal medium supplemented with α-Xgal.

Y2H screening of B cryptic species cDNA library with CP of CuLCrV as bait resulted in identification of diploid yeast colonies developing on –ALTH minimal medium. Inserts within colonies included a 2,065 bp CDS (Fig. 1, OR396905). The CDS encoded a protein with a phosphodiesterase-4 N-terminal conserved region (PDE4_NCR) and a cAMP-specific 3’,5’-cyclic nucleotide phosphodiesterase (PDEase_I) domain. BLAST analysis of the nucleotide sequence revealed >99.5% similarity with cAMP-specific 3’,5’-cyclic nucleotide phosphodiesterase of *B. tabaci* (XM_019060127.1, XM_019060128.1, XM_019060129.1). Hereon, *B. tabaci* phosphodiesterase transcripts identified in this study will be referred to as PDE4. BLASTP (NCBI/FlyBase) analysis of translated amino acid sequence showed high identity with cAMP-specific 3’,5’-cyclic phosphodiesterase of diverse insects. The dunce (dnc) gene of *Drosophila melanogaster* encoding cAMP-specific 3’,5’-cyclic phosphodiesterase, isoform I (phosphodiesterase 4 subfamily), shared >74% amino acid sequence identity with the *B. tabaci* homolog. The *B. tabaci* PDE4 transcript identified in this study had additional 5´ sequences (Fig. S1) which were missing in the *B. tabaci* sequences available on GenBank, indicating that the GenBank sequences were 5´ partial.

### Validation of interaction of PDE4 with CPs of begomoviruses

#### Y2H protein interactions of PDE4 with CPs of begomoviruses

A 2,016 bp nucleotide (Fig. S1) fragment (from start to stop codon) encoding a 671 amino acid-long PDE4 protein was PCR-amplified using *B. tabaci* cDNA as template and cloned in pGADT7 to encode a fused protein with N-terminal gal4 activation domain (AD). The first 15 amino acid sequences (MQAEQGSIGELHKYH) of the translated 671 amino acid-long PDE4 protein of *B. tabaci* matched with the initial sequences of complete cAMP specific 3’,5’-cyclic nucleotide phosphodiesterase proteins of aphids (XP_026814909.1, XP_027852822.2), thrips (XP_034240939.1), wasps (XP_044579040.1, XP_034946957.1), and beetles (XP_008198075.1, XP_018320283.1), indicating that a full-length gene of PDE4 was used in this study. The gal4 AD-PDE4 construct was screened against gal4 BD-CP (CuLCrV/SiGMV/TYLCV) or empty pGBKT7 by one-to-one Y2H mating analysis. Diploid yeast colonies containing gal4 AD-PDE4 and gal4 BD-CP constructs of the tested new-world viruses (CuLCrV and SiGMV) turned blue (Fig. 1A) on stringent minimal medium (−ALTH + α-Xgal), whereas colonies with TYLCV CP were observed (Fig. 1B) only on the less stringent (−LTH) medium. No growth was observed for yeast containing PDE4 and empty pGBKT7 constructs in either medium (Fig. 1A and B). These results indicate that PDE4 protein of *B. tabaci* interacts with the CPs of begomoviruses.

#### Pull-down assay of PDE4 using CPs of begomoviruses

**Fig 2:**
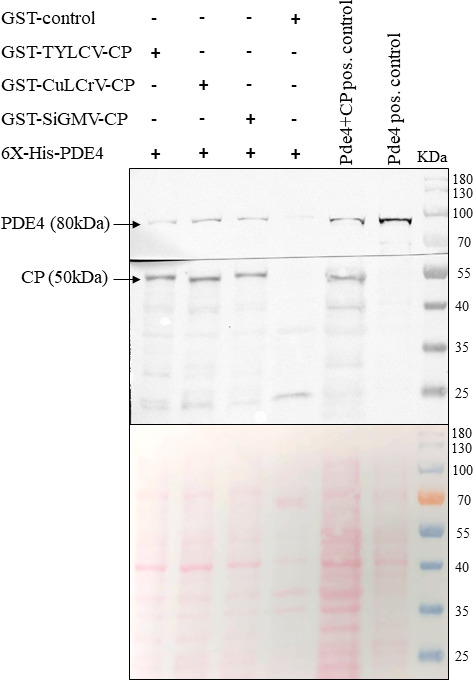
Validation of interactions between begomovirus CPs and PDE4 by pull-down assay of *B. tabaci* PDE4 protein from bacterial lysates expressing 6x-his-PDE4 by using GST-fused CPs of TYLCV, CuLCrV, and SiGMV as baits. Mixture of bacterial lysates expressing the CP and PDE4 was used as samples, and only PDE4 protein was used as positive control for detection. Monoclonal anti-GST and anti-6x-his antibodies were used to detect CPs and PDE4, respectively. The ponceau-stained membrane shows the protein amounts of the individual lanes.

Interactions between the begomovirus CPs and the PDE4 protein were validated by GST pull-down assay using CPs of CuLCrV, SiGMV, and TYLCV expressed as fused proteins (baits) with N-terminal GST to capture expressed 6x-his-PDE4 proteins from soluble fractions of the bacterial lysate. Results showed that GST-fused CPs of CuLCrV, SiGMV, and TYLCV bound to PDE4 of *B. tabaci,* whereas no binding was detected between GST and PDE4 (Fig. 2).

#### PDE4—begomovirus CP interaction in the whitefly midgut

**Fig 3:**
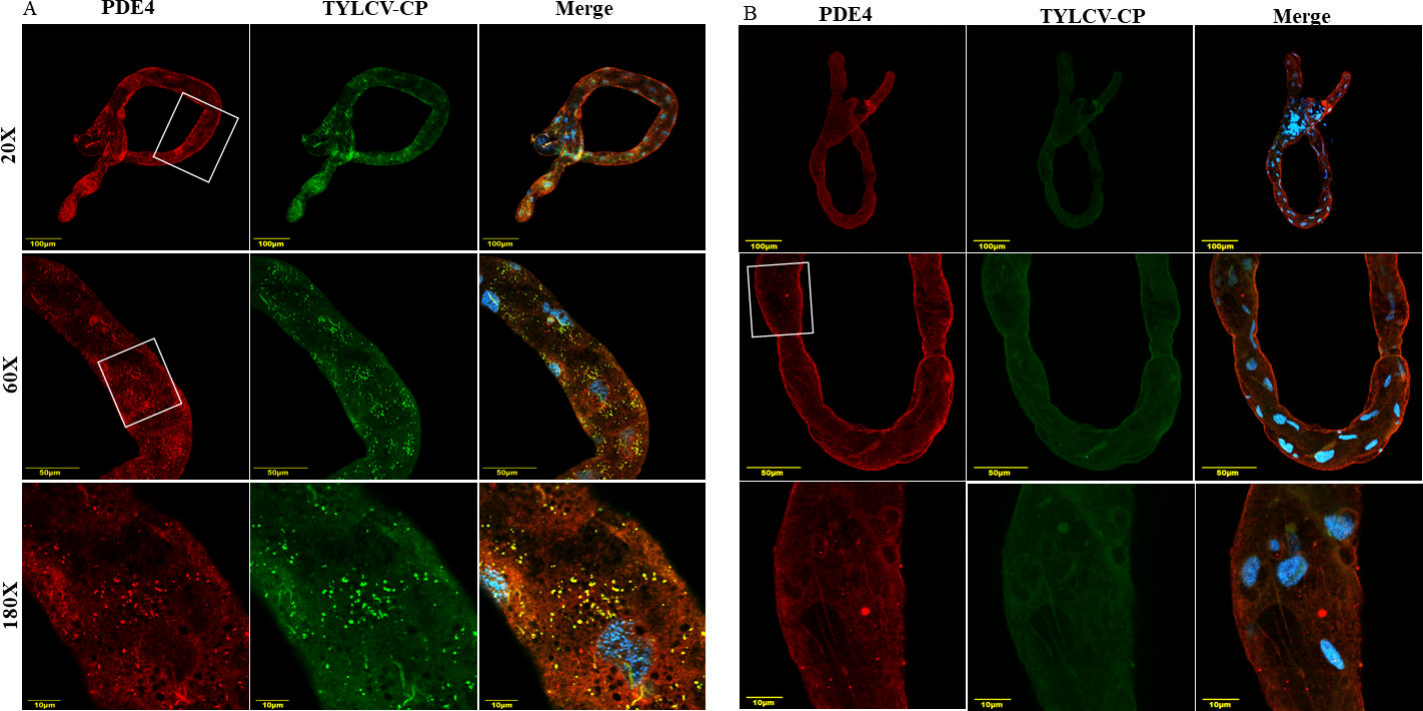
Localization of PDE4 protein and TYLCV in the midgut of (**A**) viruliferous and (**B**) non-viruliferous *B. tabaci* reared on TYLCV-infected and non-host cotton plants, respectively. TYLCV was detected using polyclonal anti-TYLCV CP antibody and cy5 (green) conjugated secondary antibody. PDE4 was detected using polyclonal anti-PDE4 antibody and cy3 (red) conjugated secondary antibody. The overlay of green and red channels is indicated in yellow, and midgut cell nuclei, stained with DAPI, are indicated in blue. The gut area magnified to 60× and 180× are depicted in white square boxes.

The midgut of *B. tabaci* is a critical tissue organelle wherein multiple proteins interact with begomoviruses to facilitate passage of the virus across the gut barrier into the hemocoel. To know whether PDE4 and begomoviruses co-localize within the whitefly midgut, PDE4 proteins were detected in the midgut (Fig. 3A) of viruliferous (TYLCV) and non-viruliferous (Fig. 3B) *B. tabaci* adults using polyclonal anti-PDE4 antibody. Whitefly PDE4 protein co-localized with TYLCV in viruliferous whitefly midgut. The co-localization appeared in the cellular cytoplasm around the nuclei (60/180× magnification), while in the negative control, using only secondary antibodies did not show any localization of either protein (Fig. S2).

### Alteration of PDE4 expression and cellular cAMP in whitefly adults post virus acquisition

#### Quantitation of PDE4 mRNA and protein post virus acquisition by qPCR and western blot

**Fig 4:**
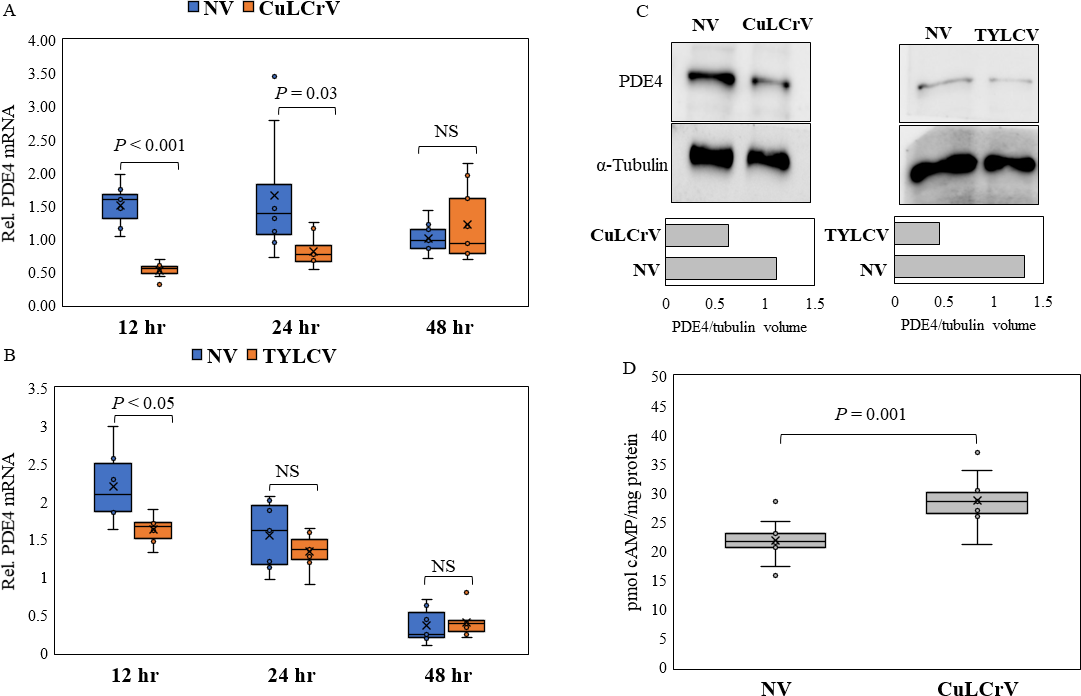
Relative quantitation of PDE4 mRNA in viruliferous whitefly adults post 12, 24, and 48 hours of AAP on (**A**) CuLCrV- or (**B**) TYLCV-infected squash/tomato plants compared with non-viruliferous whitefly (NV) adults exposed to non-infected plants. (**C**) Abundance of PDE4 protein detected by western blot analysis of soluble proteins extracted from viruliferous whitefly adults post 12 hours of virus acquisition (CuLCrV/TYLCV) compared with non-viruliferous (NV) adults. PDE4 band volumes (intensity) normalized to α-tubulin protein bands of viruliferous (CuLCrV/TYLCV) and NV control samples are depicted in bar graphs. (**D**) Quantitation of cAMP (picomoles per milligram whitefly protein) in viruliferous (post 12 hours of CuLCrV acquisition) and non-viruliferous (NV) whitefly adults.

To understand the role of PDE4 post virus acquisition, we compared the expression of PDE4 (mRNA/protein) between viruliferous (CuLCrV/TYLCV) and non-viruliferous whitefly adults. Relative abundance of PDE4 mRNAs was compared using qPCR between viruliferous and non-viruliferous whitefly adults at different time periods (12, 24, and 48 hours) post virus acquisition. PDE4 mRNA levels were significantly reduced in whitefly adults post 12 and 24 hour acquisition of CuLCrV (Fig. 4A), but not in adults post 48 hours of acquisition. Similarly, PDE4 mRNA levels were significantly reduced only in whitefly adults post 12 hour acquisition access on TYLCV-infected plants (Fig. 4B). Moreover, PDE4 protein expression was compared between viruliferous (CuLCrV/TYLCV) and non-viruliferous whitefly adults 12 hours post virus acquisition by western blotting. The PDE4 protein expression was reduced in both CuLCrV and TYLCV viruliferous adults compared with non-viruliferous whitefly adults (Fig. 4C) with higher normalized PDE4 band volumes (intensity) in non-viruliferous compared with viruliferous adults.

#### Measurement of cellular cAMP on virus acquisition

To confirm whether reduced expression of PDE4 on virus acquisition directly results in higher cellular cAMP levels, cAMP concentrations were quantitated in non-viruliferous and viruliferous (CuLCrV) whitefly adults 12 hours post-acquisition access to virus-infected or non-infected plants by cAMP-specific ELISA. Viruliferous whitefly samples (*N* = 12) 12 hours post virus (CuLCrV) acquisition contained significantly higher cAMP levels compared with non-viruliferous adults (Fig. 4D).

### Retention and transmission of begomoviruses are directly affected by cAMP levels within *B. tabaci*

#### Inhibition of PDE4 by rolipram

**Fig 5:**
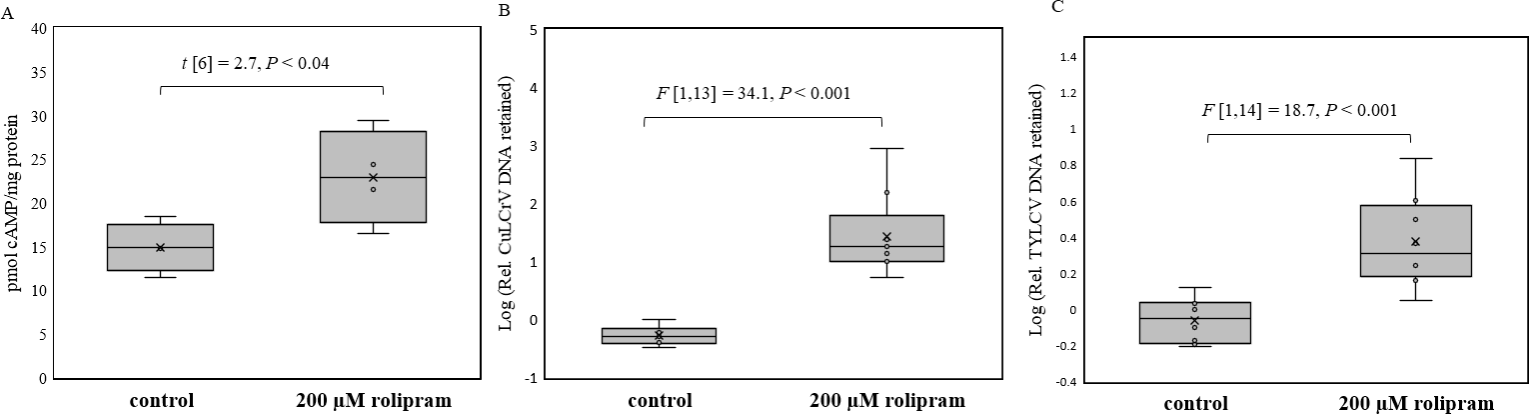
(**A**) Quantitation of cAMP (picomoles per milligram whitefly protein) in *B. tabaci* adults fed for 48 hours on 20% sucrose diet with either 0.8% ethanol (control) or 200 µM rolipram, an inhibitor of PDE4. (**B**) Relative quantitation of CuLCrV DNA and (**C**) TYLCV DNA retained (normalized to the β-tubulin gene of the whitefly) in whitefly adults post virus acquisition (24 hours) from infected squash/tomato plants and overnight gut clearing when pre-fed on 20% sucrose diet (48 hours) with 0.8% ethanol (control) or with 200 µM rolipram.

Sucrose diet supplemented with rolipram (200 µM) was fed to whitefly adults for 48 hours to inhibit PDE4 and elevate intracellular cAMP. Mean cAMP concentration quantitated by competitive ELISA was significantly higher (*P* = 0.038, *t* = 2.65, df = 6) in *B. tabaci* adults feeding on rolipram-supplemented diet compared with adults on the control diet (Fig. 5A).

#### Modulation of cAMP by feeding rolipram or SQ22536 alters begomovirus retention in whitefly adults

The effect of increased cAMP on begomovirus retention was investigated by comparing virus retention by qPCR in whitefly adults pre-fed (48 hours) on either rolipram (200 µM) or control diet and allowed an AAP (24 hours) on CuLCrV/TYLCV-infected plants. Whitefly adults that fed on rolipram diet prior to virus acquisition from infected plants retained significantly higher quantities of CuLCrV (Fig. 5B) and TYLCV (Fig. 5C) compared with adults that fed on control diet.

**Fig 6:**
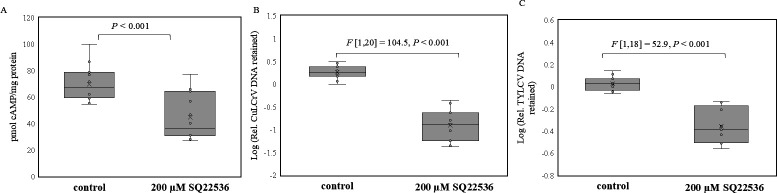
(**A**) Quantitation of cAMP (picomoles per milligram whitefly protein) in *B. tabaci* adults fed for 48 hours on 20% sucrose diet with 0.8% ethanol (control) or with 200 µM SQ22536, an inhibitor of adenylyl cyclase. Relative quantitation of (**B**) CuLCrV and (**C**) TYLCV DNA retained (normalized to the β-tubulin gene of the whitefly) in *B. tabaci* adults post virus acquisition (24 hours) from infected squash/tomato plants and overnight gut clearing when pre-fed on 20% sucrose diet (48 hours) with 0.8% ethanol (control) or with 200 µM SQ22536.

Similarly, to know the effect of decreased cAMP concentrations on begomovirus retention, whitefly adults were treated with SQ22536, an inhibitor of AC that catalyzes conversion of ATP to cAMP. Begomoviruses retained in whitefly adults pre-fed (48 hours) on 20% sucrose diet supplemented either with SQ22536 or 0.8% ethanol (control) post virus acquisition (24 hours) from CuLCrV/TYLCV-infected plants and gut cleared were quantitated by qPCR. Whitefly adults that fed on diets containing SQ22536 (200 µM) had significantly reduced cAMP levels (Fig. 6A) and retained reduced quantities of CuLCrV (Fig. 6B) and TYLCV (Fig. 6C) compared with adults on the control diet post virus acquisition and overnight gut clearing.

#### Gene silencing of PDE4 and AC alters begomovirus retention

**Fig 7:**
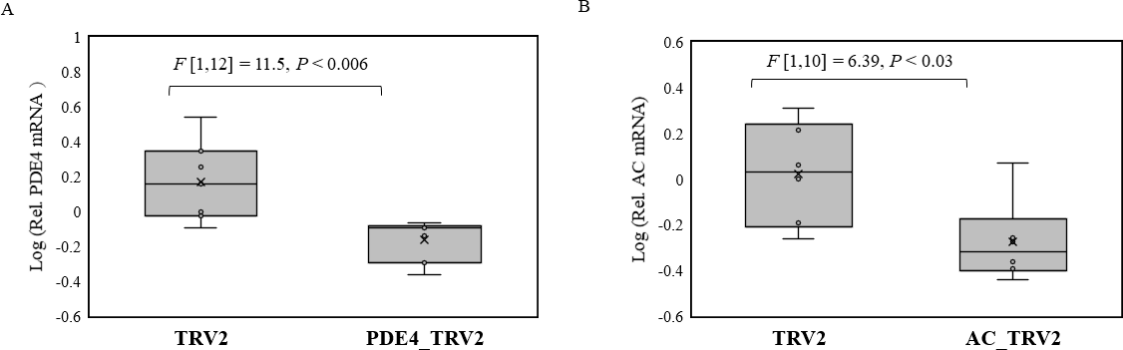
Relative quantitation of mRNA (normalized to the β-tubulin gene of the whitefly) of (**A**) PDE4 or (**B**) AC in F1 *B. tabaci* adults reared on tomato plants transformed with PDE4_TRV2 or AC_TRV2 constructs compared with TRV2 control plants.

**Fig 8:**
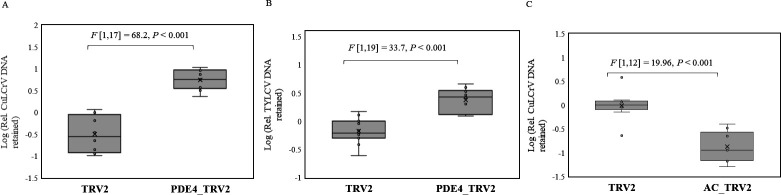
Relative quantitation of (**A**) CuLCrV and (**B**) TYLCV DNA retained (normalized to the β-tubulin gene of the whitefly) in F1 *B. tabaci* adults reared on either PDE4_TRV2 or TRV2 plants post 24 hours of virus acquisition from infected plants followed by overnight gut clearing on cotton plants. (**C**) Relative quantitation of CuLCrV DNA retained post 24 hours of virus acquisition and overnight gut clearing in F1 adults reared on AC_TRV2 compared to TRV2 control.

PDE4 and AC genes of the whitefly were silenced by continuous acquisition of PDE4 and AC-specific dsRNA from tomato plants transiently transformed with recombinant tobacco rattle virus (TRV) vectors containing PDE4 and AC gene sequences of *B tabaci*. Relative quantitation of PDE4 (Fig. 7A) and AC (Fig. 7B) mRNAs by qPCR showed significant silencing in F1 whitefly adults reared on tomato plants transformed with PDE4_TRV2 or AC_TRV2 constructs compared with control plants transformed with only TRV2. The effect of silencing PDE4 and AC genes on begomovirus retention within whitefly was quantitated by qPCR. PDE4-silenced F1 adults (PDE4_TRV2) retained higher quantities of CuLCrV (Fig. 8A) and TYLCV (Fig. 8B) compared with control (TRV2) samples 24 hours post virus acquisition from infected plants and overnight gut clearing. In contrast, AC-silenced (AC_TRV2) F1 adults retained reduced amounts of CuLCrV (Fig. 8C) than control (TRV2).

#### Transmission of CuLCrV is affected by cAMP levels within whitefly

**Fig 9:**
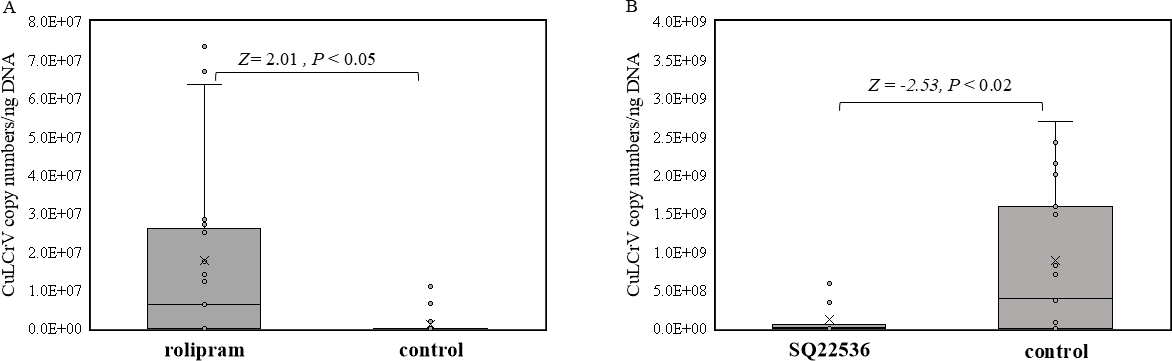
Quantitation and comparison of CuLCrV loads in infected squash plants inoculated with viruliferous *B. tabaci* adults fed on (**A**) rolipram or (**B**) SQ22536 with that of viruliferous whiteflies that fed on control diet.

To assess the effect of elevated cAMP on begomovirus transmission, whitefly adults were provided with an acquisition access for 48 hours on 20% sucrose diet with/without rolipram (200 µM) and then provided with 24 hours of acquisition access on CuLCrV-infected plants; these treated whiteflies were released to inoculate non-infected squash plants. Rolipram increased transmission efficiency (Table 2; Fig. S3) of CuLCrV by *B. tabaci* adults in all three replications. Percentage of plants infected using rolipram-fed whiteflies (93.7%) differed significantly (χ^2^ = 22.1, *P* < 0.01) from percentage of plants infected (57.8%) using control whiteflies. Similarly, to evaluate the effect of reduced cAMP on begomovirus transmission, transmission efficiency of CuLCrV was compared between whitefly adults with acquisition access on a diet with and without SQ22536. Unlike rolipram treatment, no significant differences in transmission efficiency between SQ22536 (22 infected out of 25 inoculated plants, 88%) or control diet (21 infected out of 24 inoculated plants, 87.5%) fed whitefly adults were observed. Furthermore, CuLCrV loads in plants inoculated with rolipram/SQ22536 and control whiteflies were quantitated. Interestingly, virus loads in plants infected with rolipram-fed whitefly adults were significantly higher (Fig. 9A) than control. In contrast, plants infected with SQ22536-fed whitefly adults had significantly lower (Fig. 9B) virus loads compared with control.

**TABLE 2:** Transmission of CuLCrV by *B. tabaci* adults fed on rolipram or control diet and tested by PCR 15 days post inoculation^*a*^

	No. of plants infected/no. of plants inoculated			
	Replication 1	Replication 2	Replication 3	Total
Rolipram (200 µM)	19/20 (95%)	24/24 (100%)	16/19 (84.2%)	59/63 (93.7%)
Control	15/24 (62.5%)	11/20 (55%)	11/20 (55%)	37/64 (57.8%)

^*a*^ The experiment was set up in three replicates for each treatment (rolipram/control diet) with a minimum of 19 plants inoculated in each replicate.

## DISCUSSION

Whitefly transmitted begomoviruses such as CuLCrV, SiGMV, and TYLCV cause severe economic losses to vegetable production in southeastern and southwestern United States. Disruption of genes/pathways critical for virus transmission can lead to effective management of begomoviruses. In this study, a gal4-based Y2H system was used to screen the cDNA library of *B. tabaci* (B cryptic species) with the CP of CuLCrV as a bait to identify interacting whitefly proteins involved in the transmission of begomoviruses. This study identified PDE4, a cAMP-specific 3’,5’-cyclic nucleotide phosphodiesterase of the whitefly that interacts with the CPs of begomoviruses via Y2H, and confirmed the interaction through pull-down assay and co-immunolocalization in the whitefly midgut. PDE4 family of enzymes regulate intracellular cAMP, a second messenger regulating multiple downstream signaling cascades. Two enzymes, AC and cAMP-dependent phosphodiesterase, maintain the intracellular cAMP concentration. Extracellular ligand activity with cell surface receptors such as the G-protein-coupled receptor activates AC, which catalyzes the synthesis of intracellular cAMP from ATP, whereas PDE4 hydrolyzes cAMP to AMP (27). Intracellular cAMP concentrations are sensed by its binding with two downstream proteins, protein kinase A (PKA) and exchange protein directly activated by cAMP (Epac). Cyclic AMP-dependent activation or deactivation of these receptors orchestrate diverse metabolic and physiological functions of the cell. Immune response to infection with pathogenic microbes can also be modulated by intracellular cAMP concentrations (28). Elevated intracellular cAMP leads to downregulation of NF-κB-induced transcription of inflammatory and apoptotic responses or upregulation of anti-inflammatory cytokines (29).

Pathogenic viruses (30, 31) and bacteria (32) of humans are known to elevate cAMP levels within its host cell to evade immune response, and conversion of cAMP to AMP leads to heightened immune response (33). Thus, intracellular cAMP has been an important target for therapeutic purposes for years (24). The results of this study show that begomovirus acquisition causes significant reduction in PDE4 expression and higher cellular cAMP levels in whitefly adults post 12 hours of acquisition. However, the differences in PDE4 mRNA transcripts with virus acquisition incrementally diminished with longer acquisition times of 24 to 48 hours, indicating that the elevation in cAMP is an initial response to virus acquisition. Multiple studies in lepidopteran insects have shown that elevated cAMP can impair insect immunity and cellular response to infection (34, 35). Interestingly, our results conclusively prove that elevation of cAMP in whitefly by inhibition of PDE4 causes increased begomovirus retention and transmission. Similarly, reduction in cAMP levels by inhibiting AC led to decreased begomovirus retention and lower virus loads in infected plants after transmission. It is thus plausible to conclude that begomoviruses interact with the whitefly PDE4 to elevate cellular cAMP levels at initial stages of acquisition to facilitate its circulative pathway inside the whitefly. However, the exact mechanism of how elevated cAMP facilitates virus retention and transmission remains unknown.

The findings of this study indicate potential for using insecticides/chemicals that reduce cAMP levels in the whitefly for management of begomoviruses. Bio-pesticides modulating intracellular cAMP of insects by activation of AC or inhibition of PDE have been used as insecticides previously (36). Further exploration and exploitation of such natural active ingredients can open new avenues for alternative and sustainable management of begomoviruses. Recent novel strategies, such as foliar application of nano-clay particles loaded with dsRNA targeting virus and insect genes, have been shown to be effective for the management of plant viruses (37) and the whitefly vector (38). However, management of insect-transmitted viruses by foliar dsRNA applications can be made more effective if the insect vector and virus transmission mechanism are simultaneously targeted. Aiming to decrease cAMP levels of the whitefly by targeting the AC gene through foliar application of RNAi insecticides can be an effective strategy to reduce begomovirus epidemics and will be investigated in the future.

## Data Availability

The complete gene sequence of *B. tabaci* PDE4 has been deposited in NCBI under accession number OR396905. Raw data files for all figures are available in File S1 in the supplemental material.

## SUPPLEMENTAL MATERIAL

Supplemental figures jvi.02172-24-s0001.docx Figures S1 to S3.

File S1 jvi.02172-24-s0002.xlsx Raw data.

ASM does not own the copyrights to Supplemental Material that may be linked to, or accessed through, an article. The authors have granted ASM a non-exclusive, world-wide license to publish the Supplemental Material files. Please contact the corresponding author directly for reuse.
