# Supplementary material for: Begomovirus capsid proteins interact with cyclic adenosine monophosphate (cAMP)-specific phosphodiesterase of its whitefly vector and modulate virus retention within its vector
Source: J Virol. 2025 Feb 11;99(3):e02172-24. doi: 10.1128/jvi.02172-24 (PMC11915853; doi:10.1128/jvi.02172-24)

**Supplementary file S1:** The Excel file shows the raw data used for Fig. 4-9.

**Fig. S1:** Nucleotide sequence of the PDE4 construct identified by Y2H screening of *B. tabaci* (B cryptic species) cDNA library against CP of CuLCrV. Nucleotide sequences of the smart III oligo is denoted in red, primers used for PCR amplification of PDE4 (2016 bp) for Y2H mating/pull-down assay, qPCR (182 bp) and dsRNA (557 bp) TRV2-construct are highlighted in blue, grey, and yellow, respectively. Bold letters denote start and stop codons of the PDE4 gene used in this study.

**Figure S2:** Midguts isolated from viruliferous (TYLCV) whitefly adults that were fixed, permeabilized and incubated with cy3 (green) and cy5 (red) conjugated secondary antibody without the primary antibody and used as negative control.

**Fig. S3:** Transmission efficiency of CuLCrV by *B. tabaci* adults fed on rolipram or control diet were tested by PCR, 15 days post inoculation. The experiment was set up in three replicates for each treatment (rolipram/control diet) with a minimum of 19 plants inoculated in each replicate. The plant samples that tested positive for CuLCrV by PCR are marked in white numbers and the negative samples are marked in red. A positive (+) control DNA isolated from an CuLCrV and no template control (ntc) were used for the PCR test in each replicate.


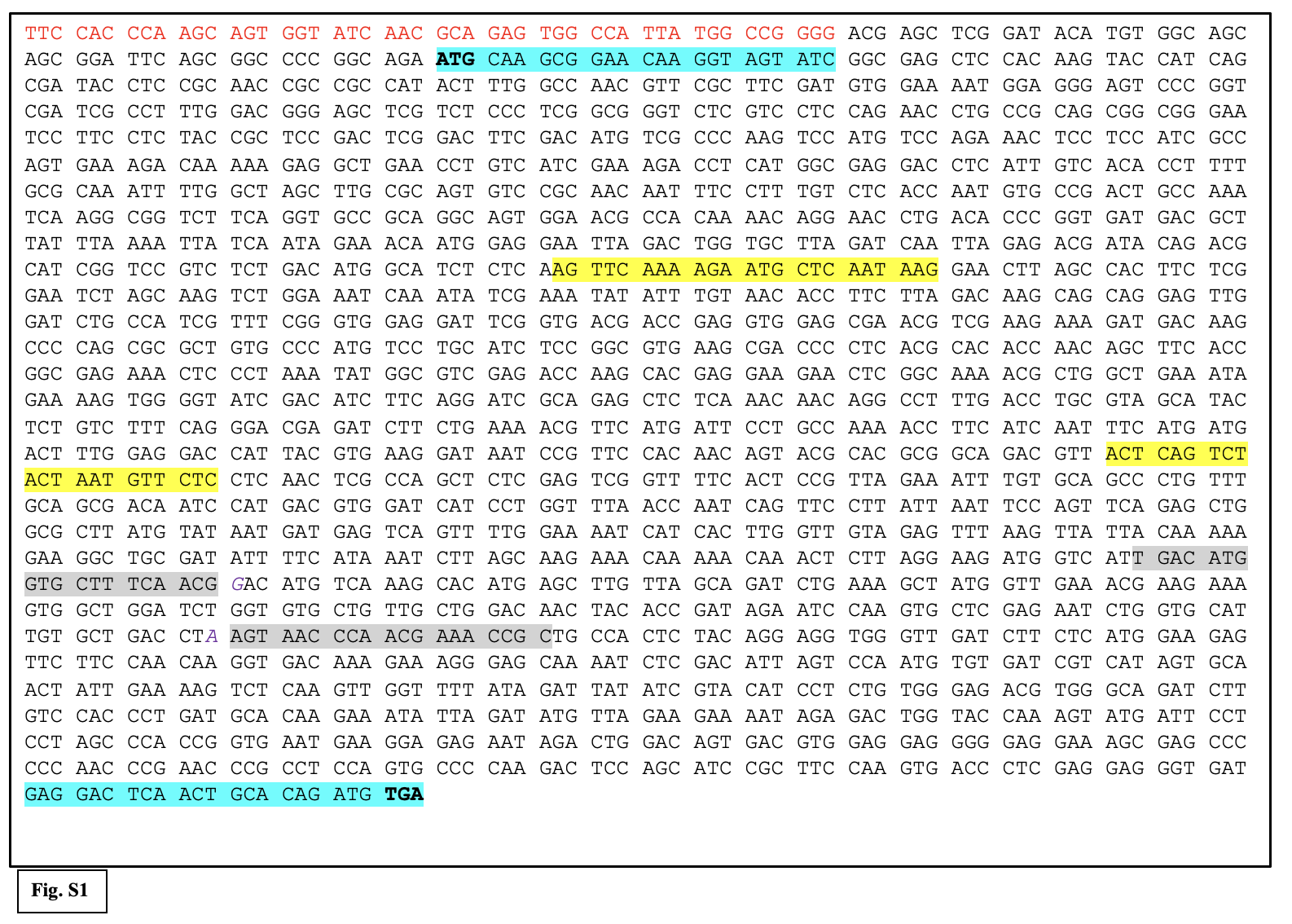


**
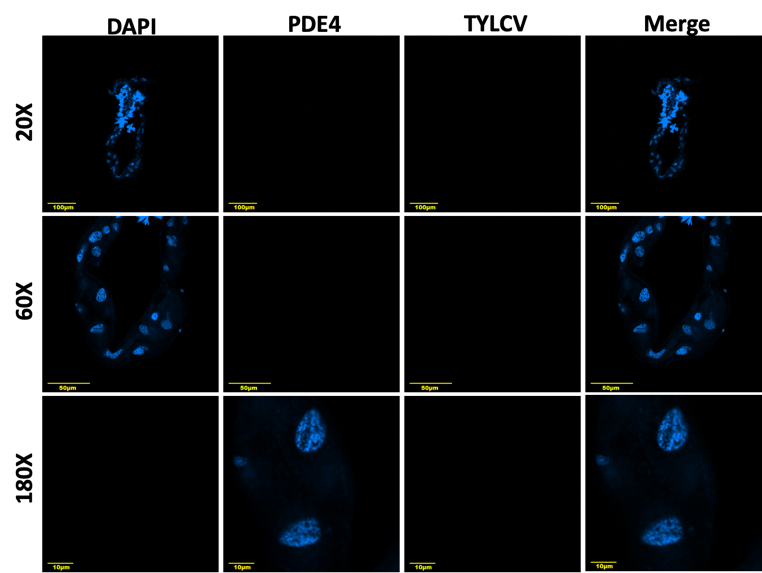
**

**Fig. S2**


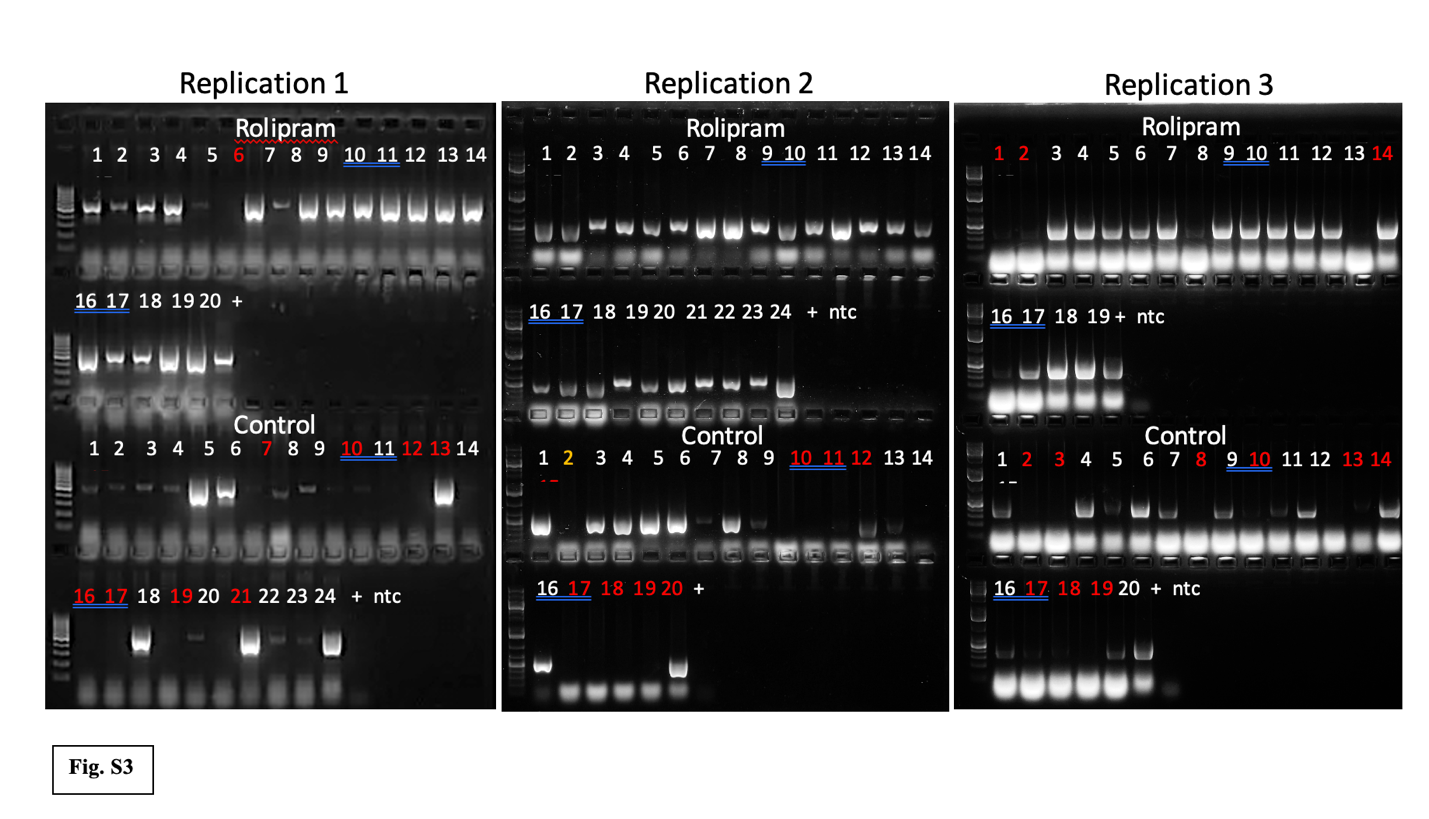

Supplement: Supplemental figures — Figures S1 to S3. [file jvi.02172-24-s0001.docx]
